# Surface Expressions of Subsurface Sediment Mobilization Rooted into a Gas Hydrate-Rich Cryosphere on Mars

**DOI:** 10.1038/s41598-019-45057-7

**Published:** 2019-06-13

**Authors:** Barbara De Toffoli, Riccardo Pozzobon, Matteo Massironi, Francesco Mazzarini, Susan Conway, Gabriele Cremonese

**Affiliations:** 1 Department of Geosciences, University of Padova, Via Gradenigo 6, Padova, 35131 Italy; 2 INAF, Osservatorio Astronomico di Padova, Vicolo dell’Osservatorio 3, Padova, I-35122 Italy; 3 Istituto Nazionale di Geofisica e Vulcanologia, Via Della Faggiola 32, Pisa, 56100 Italy; 4 Laboratoire de Planetologie et Geodynamique, Universite de Nantes, CNRS UMR, 6112 Nantes, France

**Keywords:** Structural geology, Geomorphology, Astrobiology

## Abstract

We report on evidence for fluid circulation in the upper crust of Mars, which could create environments favorable for life and its development. We investigate the nature of the thumbprint terrains covering part of Arcadia Planitia in the Martian northern hemisphere. Our analytic procedure allowed us to (i) hypothesise a potential relationship between these thumbprint terrains and an inferred underground fracture network that extends to where the clathrate-rich cryosphere contacts with the underlying hydrosphere; (ii) support the hypothesis that these thumbprint terrains are made of fine grained loosely packed materials erupted from deep beneath the subsurface mobilized by water; and (iii) date the thumbprint terrains of Arcadia Planitia to ~370 Ma. We conclude that the study area is an area worthy of astrobiological investigation, bringing water and fine grained sediment from depth to the surface for investigation.

## Introduction

**Figure 1:**
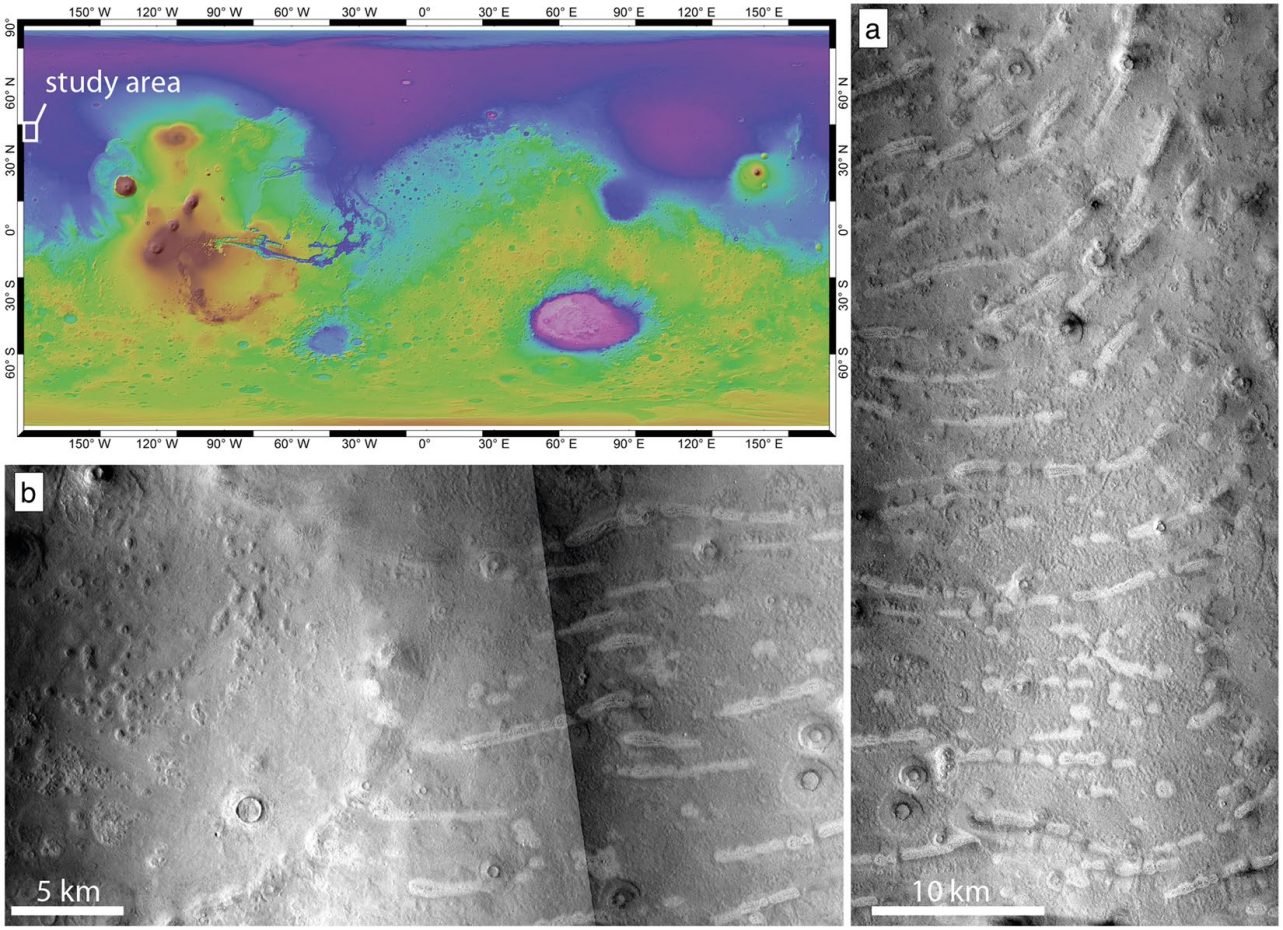
On the map: location of the study area is marked by the white box. (**a**,**b**) Examples of the characteristic morphology of the thumbprint terrain (CTX: F03_036957_2301; CTX: P21_009055_2314; mosaics generated through https://pilot.wr.usgs.gov)^62^.

The northern lowlands of Mars are thought to have hosted large bodies of water in the geological past and previous studies have proposed various timings and timescales for the lifetimes of these bodies. Visible contacts encircling the northern lowlands have been interpreted as paleoshorelines (Fig. 1) and suggest the existence of an ocean during the Early Noachian Epoch (until ~4 Ga) that covered one third of the planet^1^. This ocean was hypothesised to be in equlibrium with relatively warm and wet environmental conditions prevailing at the time. Later in the Hesperian (until ~2 Ga)^1^ the environment of the planet is thought to have become drier. Yet, short-lived water bodies are thought to have been emplaced on the northern plains during this time by catastrophic outflow events. These water bodies likely experienced rapid freezing and sublimation after their emplacement^2^.

Our study area is in the northern lowlands, or “Vastitas Borealis”, and is located at mid-high latitudes, centered at 47°N 184°E, close to the central portion of Arcadia Planitia at around 4 km below the mean Martian elevation (Fig. 1). The geological units underlying the study area are called the Vastitas Borealis marginal and interior units, and are interpreted to be the product of pervasive alteration during the Early Amazonian of sediments emplaced during outflow events in the Late Hesperian. These outflow events are thought to be sourced from both the lowlands and highlands, and their deposits are sporadically covered by ejecta blankets from Amazonian craters^3^.

In this area, pervasive kilometre-scale positive relief morphologies have been reported, referred to as mounds or, on larger scale, as knob fields, knobby terrain or thumbprint terrain^4–7^. Thumbprint terrains (TPT) are characterised by sets of curvilinear features made up of continuous and discontinuous alignments of pitted domes. They are recognisable in several locations on the Northern hemisphere^3,8^ with Arcadia Planitia amongst the main sites^2,4,9,10^. The interpretation of these landforms is still widely debated, suggestions include magmatic volcanism^11–14^, mud volcanism^15–18^, ice-related processes^6,8,19–21^ and tsunami deposits^22,23^.

Herein we combine geomorphological information, absolute model ages and the spatial distribution analysis of the TPT alignments and produce a consistent interpretation of the these features.

## Results

### Image analyses

**Figure 2:**
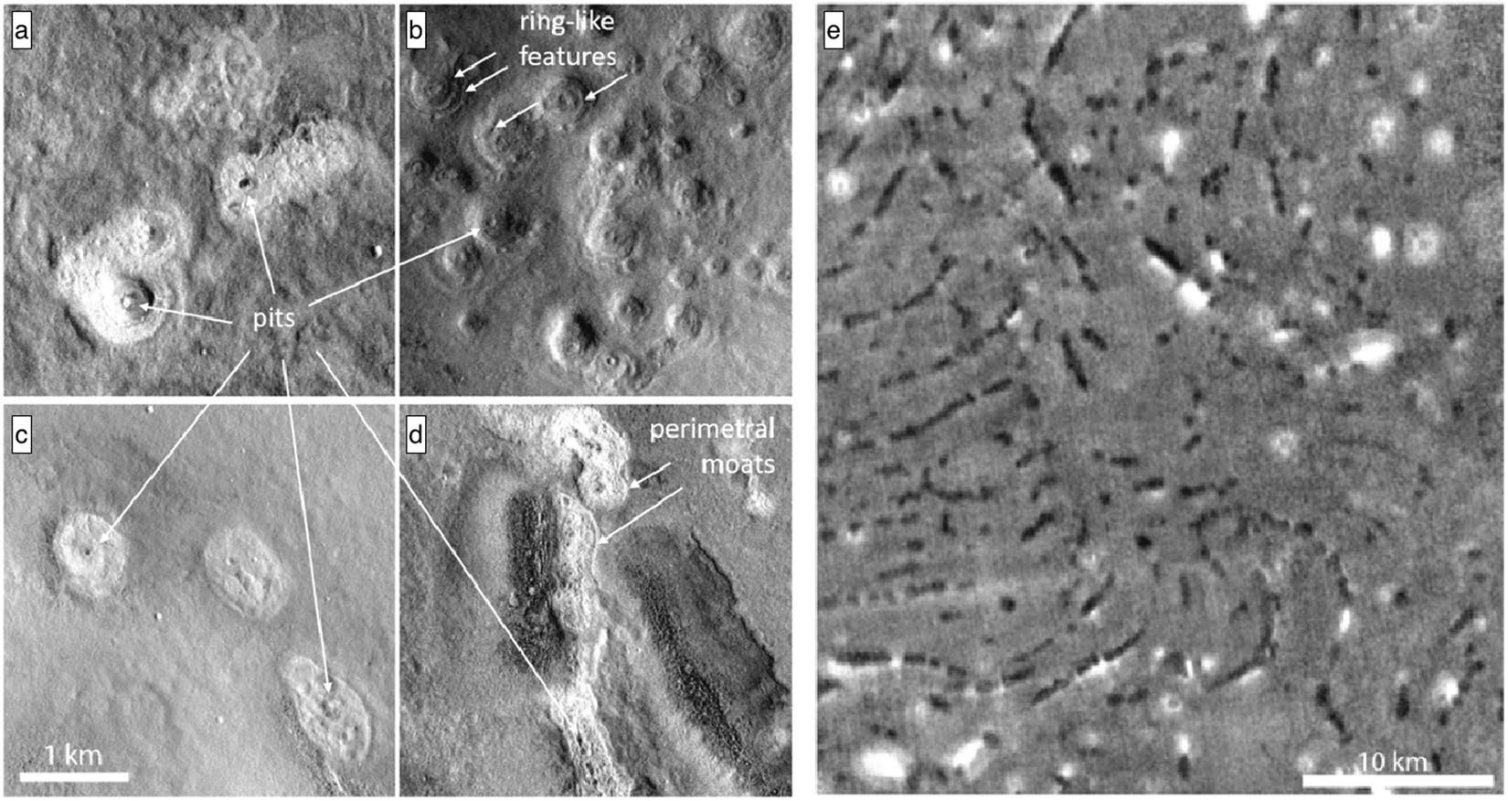
Key traits of the Low Elevation and High Albedo Features, LEHAF (**a**–**d**). Key characteristics are: relatively high albedo, higher surface roughness, oval to round basal shape, pitted summits with circumferential troughs (or ring-like features) and perimeter moats and swellings. (**e**) THEMIS Nighttime IR image where the chains of LEHAF, also shown in Fig. 1, are visible as dark patches, recognizable because of their distinctive spatial organization. (images obtained through https://pilot.wr.usgs.gov)^62^.

In the Arcadia region we found low elevation mounds that are distinctly higher albedo compared to their surroundings and are characterized by perimeter moats and swellings, a rougher surface than their surroundings and a central pit surrounded by concentric troughs (Fig. 2a,c,d). We define these objects as Low Elevation and High Albedo Features (LEHAF) (Fig. 1a,b).

The LEHAF are around 700 to 300 m in diameter, are round to oval with irregular, and/or indistinct margins which are not always readily distinguishable from their surroundings.

The large majority of the LEHAF are organized in tight arcuate chains (Figs 1 and 2d), where each individual feature frequently overlaps with adjacent one(s) to form ridges. These ridges are on average 5 km long, although the lengths range between tens of kilometers to hundreds of meters. Moats surrounding the pits can often merge into continuous elongated moats around the ridge. LEHAFs that make up the curvilinear ridges constitute the majority of the dome alignments that, by definition, characterize TPT^4^. Such ridges are distributed in arcuate parallel sets forming a pattern resembling overlapping fans.

Nighttime Infrared (IR) data from the Thermal Emission Imaging Spectrometer (THEMIS) on Mars Odyssey were also examined. The arcuate chains of the TPT are easily recognizable in the Nighttime IR THEMIS images as prominent dark linear and arcuate structures (Fig. 2e) representing a common low thermal inertia^24,25^.

**Figure 3:**
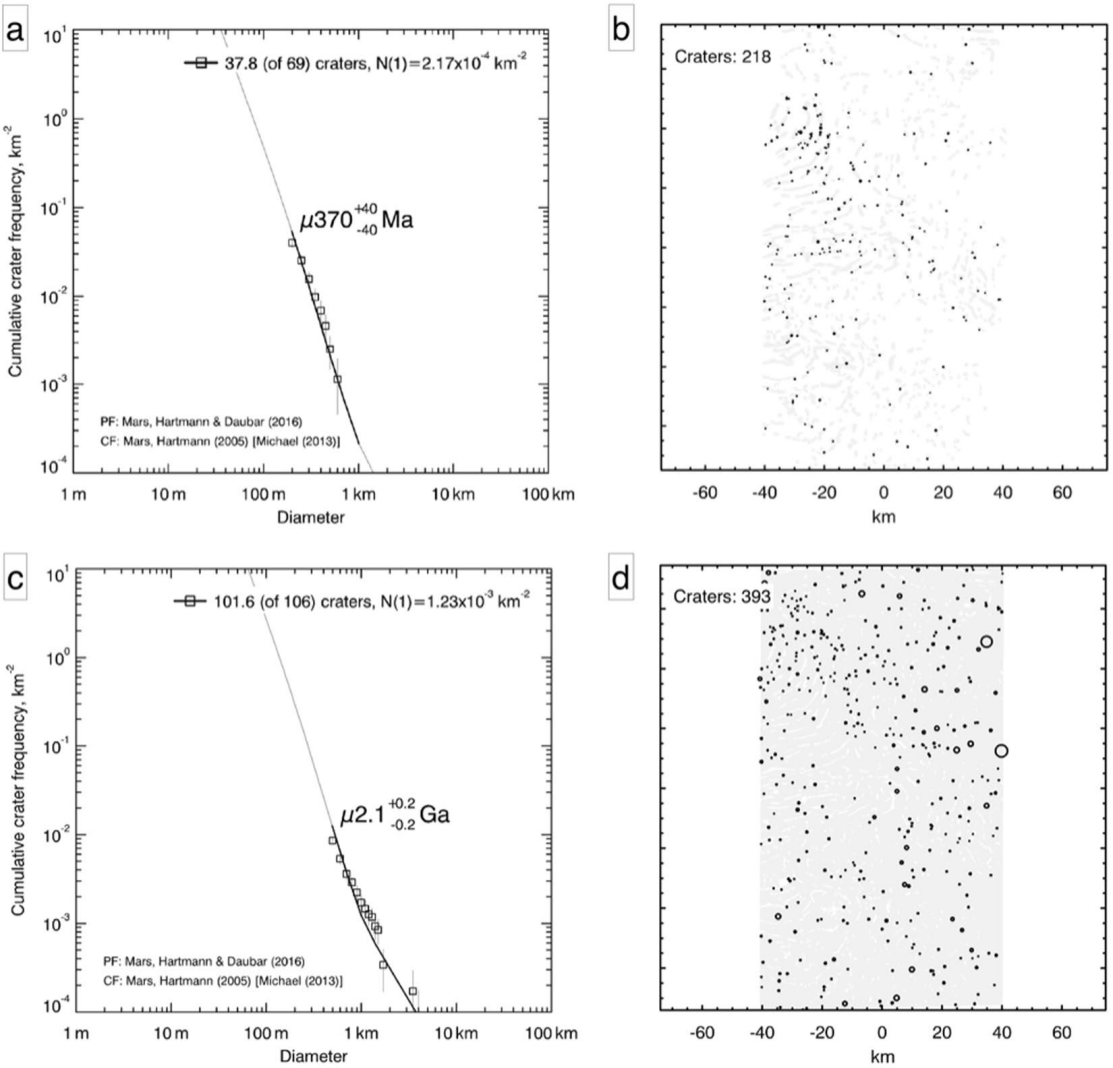
Absolute model ages determined by CSFD measurements of the TPT (**a**) and the underlying surface (**c**). The spatial distributions of the crater populations (black circles) for the analyzed surfaces (in grey): TPT (**b**) and the underlaying surface (**d**).

Statistical analysis of ﻿crater size-frequency distributions (CSFD) of impact craters on planetary surfaces is a well-established method to derive absolute ages on the basis of remotely-sensed image data^26^. CSFD-analysis was performed on the entire LEHAF assemblage as a single population of TPT, resulting in an estimated age of 370 ± 40 Ma and of 2.1 ± 0.2 Ga for the underlying surface (Fig. 3). Although it is very likely that multiple populations of LEHAF could have arisen at different times, determining the model age on the TPT as a single unit at least allows us to constrain the last occurrence of the phenomenon. There is a lack of morphological evidence to consistently discriminate the relative timing of the formation of different groups of LEHAF.

### Fractal analysis

We performed fractal analysis on the LEHAF comprising the TPT in order to understand whether their spatial patterns were consistent with a control by an underlying facture network^27^. We first extracted four clusters of LEHAF from the initial mapped LEHAF, and these clusters were extracted from the initial dataset by applying an agglomerative hierarchical clustering method^28^.

**Figure 4:**
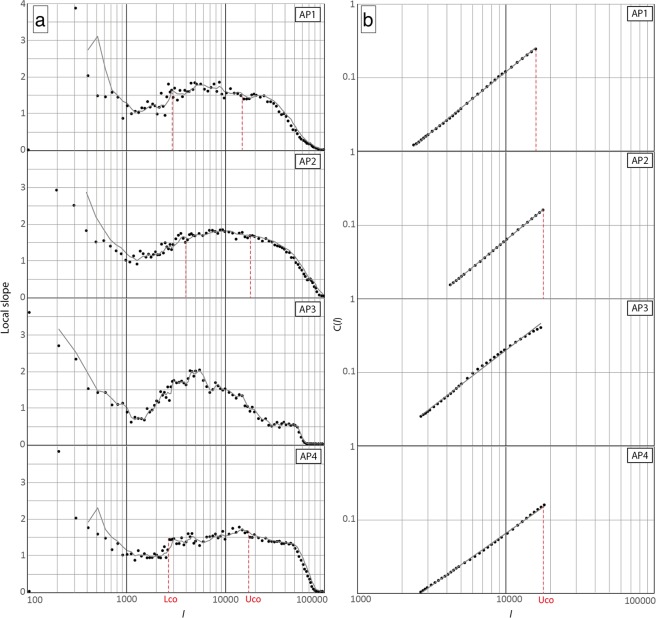
The size range of potentially interconnected fractures are represented by the plateau stage of the curve, where present, for all the identified clusters of LEHAF (**a**). Where the slope breaks after the plateau the LEHAF distribution ceases to be fractal. The corresponding *log(l)* value represents the maximum thickness of the fractured medium that connects the surface to the subsurface (Uco). (**b**) The distribution of LEHAF compared to a Power Law (solid line) for values between Lco and Uco. The goodness of the Uco value picked in the previous graph is tested. A fractal distribution should follow a Power Law. Where data matches the solid line the distribution of LEHAF/fractures is fractal. *l*: fracture length [m]; *C(l)*: correlation integral defines the correlation between point at a distance lower than *l*; Local Slope: represents the *ΔLog (C(l))/ΔLog(l)* ratio].

We applied fractal analysis to four different clusters of LEHAF in Arcadia Planitia (AP), please refer to the methods section for details. The upper cutoff (Uco) identified in this analysis provides an estimate of the depth of the fluid source^29^ and thus insight into the thickness of the fractured medium between the source and the surface in the studied region. The value of Uco for each cluster was derived from *local slope vs. log(l)* and *C(l) vs. l(m)* diagram shown in Fig. 4. The selection of the cutoffs was performed identifying the widest size range represented by the largest possible plateau in the *local slope vs. log(l)* graph^29^.

Among the identified clusters of Arcadia Planitia, three out of four show fractally distributed LEHAF. The output from cluster AP3 was too noisy and with no clear distribution pattern so, for this reason, no deductions were made from this cluster. Uco values are: 16 ± 2 km for AP1, 18 ± 2 km for AP2 and 20 ± 3 km for AP4 (Fig. 4), which also displays a second shallow plateau with Uco at 2.5 ± 0.3 km.

## Discussions

The origin of TPT is still debated and no agreement has been reached on its origin. For this reason we wanted to apply a new approach in order to shed new light on the topic. Specifically, we implemented cluster and fractal analyses which takes into consideration the interplay between the surface and the subsurface in defining the pattern of features observed at the surface.

The majority of the LEHAF appear to be circular features merged in coalescent chains and we observed summit (and also peripheral in places) pits with several concentric annular structures suggesting an eruptive mechanism^16,30–32^. The albedo of the LEHAF depends on the nature of the extruded material. Context Camera (CTX) mosaics displayed a population with an albedo higher than or equal to their surroundings while in the Nighttime IR data the same LEHAF appear darker than their surroundings due to their low thermal inertia. Thermal inertia is the ability of the subsurface to conduct and store heat energy during exposure to sunlight and to return that heat energy to the surface during the night. Thermal inertia depends on the nature of the material and can be used to identify some physical characteristics of the material. Fine grained and loosely packed materials typically have a low value of thermal inertia, while higher thermal inertia values are commonly associated with rocky terrain and exposed bedrock. Therefore, considering the morphological appearance and low thermal inertia of the LEHAF, they are likely to be composed of fine-grained loose sediments sourced from depth^16,24,33–35^.

This hypothesis would make the LEHAF of astrobiological importance because, on Earth, recurrent sediment ejections associated with liquid water are closely associated with degassing phenomenon, including methane release^35–38^ - two key life-detection proxies in the search for life beyond Earth^37–40^.

**Figure 5:**
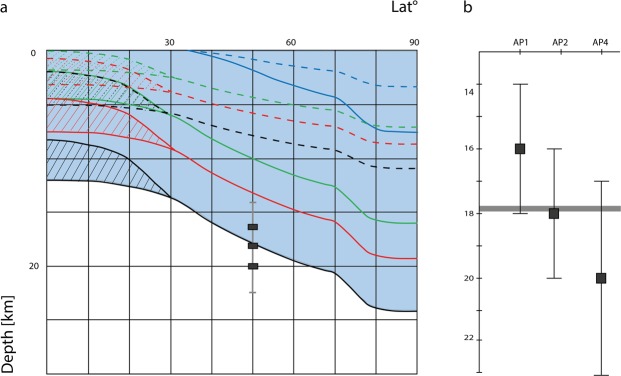
The latitudinal variation in the depth of a hydrate-rich cryosphere for three different groundwater freezing temperatures: 203°K corresponding to sulfate-rich and Mg(ClO_4_)_2_ brines (blue line); 252°K corresponding to NaCl brine (green line); 273°K corresponding to pure water (red line); 303°K corresponding to the base of the gas hydrate stability zone (black line); assumed heat flow is 15 mW m^−2^ for the solid lines and 30 mW m^−2^ for the dashed lines. Below 30° latitude, high and low regolith porosity can influence the isotherm distribution as shown by the divergent pattern^41^. Maximum extensions of fracture systems inferred beneath the TPT in Arcadia Planitia is plotted as three boxes at the latitude of Arcadia Planitia (**a**). Latitudinal break down for the maximum extension value (Uco) for each cluster (**b**). Grey line in graph b displays the gas hydrate stability zone base level taken from a.

The geomorphological evidence pointing towards sediment mobilization and eruption as the formation mechanism behind the Arcadia LEHAF is supported by the outcome of the fractal analysis. This analysis implies a link between these object fields and an underlying network of fractures tapping a reservoir at depth^27^. This in turn implies fluid circulation in the Martian upper crust when these features formed, which according to the CSFD analysis was as recently as ~ 370 Ma. Thus, we compared our estimated thickness of the fractured medium, i.e. the location at depth of the fluid source, with the estimated recent distribution of ice and water in the subsurface as given by Clifford *et al*.^41^. The Martian cryosphere is thought to be a natural water trap in the subsurface where groundwater persists in a liquid phase when its inventory exceeds the actual pore volume of the cryosphere leading to an accumulation of water in the lower portion of the crust^41,42^. In our case study, the considered region is situated in a low topographic area where an actual physical contact between the cryosphere base and the groundwater reservoir may occur when the H_2_O inventory is sufficient^1,41,42^. Brines change the proposed structure of the cryosphere because, different brines have different phase transition temperatures and pressures. Depending on the brine and its concentration, the basal limit of the cryosphere stability zone would change as shown in Fig. 5. We only show the best-insulated scenario modelled by Clifford *et al*.^41^, i.e. describing the shallowest calculated position of the isotherms marking the transition from solid to liquid phase of the brines. In this model the authors assumed a maximum realistic porosity (F = 0.35 at the surface and follows the exponential decay relationship of Clifford^42^ in the subsurface), a low thermal conductivity of the crust (0.5 W m^−1^ K^−1^) and a cryosphere fully saturated in gas-hydrates instead of water-ice. Astronomically induced variations are also described in Clifford *et al*.^41^ but, since they are estimated to have affected the cryosphere depth by just of few hundreds of meters in equatorial and polar positions, these parameters are neglected in this work.

Comparing our estimated thicknesses of the fractured medium beneath the TPT and the predicted cryosphere stability zones of Clifford *et al*.^41^, we find that almost all the mapped TPT chains are related to fracture networks extending up to 16–18 km beneath the surface which corresponds to the modelled gas-hydrate rich cryosphere-hydrosphere transition^41^. The majority of the LEHAF appear to have been fed from portions of a fractured medium that acted as a reservoir and conduit system, extending to the base of the gas hydrate stability zone. This produces a meaningful relationship between the potential gas hydrate distribution in the subsurface and the occurrence of such surface features.

This interpretation has further implications for the identified AP4 cluster of LEHAF. This cluster has a double plateau, and the shallower one (Uco ~3 km) was discarded from our analyses due to its very narrow size range leading to high statistical uncertainties^43^. However, such a plateau could be interpreted as: either the product of an earlier pulse of the same phenomenon and preserved without being completely obliterated by newer inputs of upwelling materials, or as a result of the circulation of brines with a different composition or at higher temperatures, or a combination of the two, which could result in a shallower reservoirs.

This link leads us to infer that water, ostensibly as a result of gas hydrate dissociation, plays a central role in subsurface processes that may vent fluids and sediments to the surface. This supports the hypothesis that sediment mobilization and eruption as a formation process for the TPT that we examined. Notably, crustal conditions favoring the sediment mobilization process could potentially be found across the entire northern plains, indicating that a similar phenomenon may have operated on a wider portion of the planet and therefore could explain TPT in other locations. Such conditions are hypothesized to be true for the Amazonian period^41^. In addition, our study suggests that these conditions could have persisted until even the most recent Amazonian times (at least ~370 Ma).

Although alternative interpretations of TPT are presented in the previously cited literature, we find that they do not match the observations and measurements of the TPT in our study area. The magmatic volcanism hypothesis is in contrast with the observed albedo and thermal inertia of the Arcadia mounds^16,24,33–35^, because this hypothesis predicts low albedo, high thermal inertia surface deposits. The hypothesis that the TPT originates from ice at a shallow depth, creating forms such as pingos, can be rejected due to (i) the LEHAF’s spatial arrangement strongly suggestive of control by an underlying fractal fracture network^27^; (ii) the absence of pingos’ key traits such summit fracture(s), or shallow rimmed irregular depressions as remnants of their degradation^44–46^; and (iii) the bright albedo, differentiating LEHAF from their surroundings, suggesting a material heterogeneity not required by a shallow water melting/freezing process. Lastly, the tsunami hypothesis is inconsistent with our data in several ways. Primarily, the TPT (and the underlying unit) are too young to have experienced the ocean floor environment necessary to this kind of deposit formation (~3 Ga)^22,23,62^.

## Conclusions

We report on observations and analyses that support fluid circulation in the Martian upper crust which could have involved large areas of the northern lowlands and is expressed at the surface as thumbprint terrain (TPT). We interpret these features as evidence of subsurface sediment mobilization and extrusion, because: (i) their geomorphological traits are consistent with them being chains of eruption locii; (ii) their thermal inertia is consistent with that of fine grained loose materials; (iii) their spatial patterns suggesting a control by a subsurface network of fractures acting as conduits for fluid flow from the subsurface toward the surface and (iv) the coincidence between the estimated extent of the fracture system and the estimated depth of the base of the gas-hydrate rich cryosphere. This final point makes a direct connection between the thumbprint terrain and the dissociation of subsurface clathrate deposits. Firstly, clathrates could provide an insulating layer in the subsurface and produce thermal anomalies beneath which local cryospheric melting could occur. This in turn could produce shallow groundwater, and sporadic resurgence events^47–49^, explaining the eruption of sediments and water to create TPT. Additional thinning and destabilization of the cryosphere could be induced at the ice-water contact due to a combination of freezing point depression by high salinity fluid and low temperature convection of groundwater^41,50,51^. Secondly, clathrates can act as massive storage of gasses in the subsurface^52,53^, which could be released through their destabilization via the conduits underlying the TPT.

The implication is that thumbprint terrains could therefore be gas emission centers responsible for geologically recent degassing pulses. Even if no direct relationship between TPT and any biological contribution can be drawn from this new data, such environments should be taken into account for astrobiological exploration purposes. One of the large-scale astrobiological questions concerns the possibility that life arose on other planets beyond Earth (for a complete review refer to Cockell^54^). In this work we address this aspect by widening and improving the knowledge of environments where life could find niches to survive on planet Mars. The implication that there was long-lasting fluid activity in geologically recent times under Arcadia Planitia marks a step forward in understanding the Martian environment and the possibility for the presence of life.

This study likewise suggests that LEHAF, and thus TPT, should be in focus for further geological and astrobiological investigations. They appear to be products of subsurface sediment mobilization by water (from gas hydrate dissociation). Furthermore, the erupted materials would originate at depths otherwise inaccessible to our present-day instrumentation making them a unique chance to study materials originating from several kilometers beneath the surface.

## Methods

We compiled an extensive map of LEHAF recognisable in the study area and performed geomorphological observations, cluster and fractal analyses. By the application of the following procedures we tested the potential relationship between LEHAF and underlying systems of connected fracture networks that could have acted as pathways for fluid percolation. We then dated these features by means of absolute model ages based on crater-size-frequency-distributions (CSFDs).

### Image analysis

We mapped LEHAF above 100 m in diameter. The geomorphological observations were performed on a mosaic of 46 Mars Reconnaissance Orbiter (MRO) Context Camera CTX images produced by means of USGS Astrogeology service’s Pilot software (pilot.wr.usgs.gov). The CTX images have a resolution of 6 m/pixel, and so we also used MRO High Resolution Imaging Science Experiment (HiRISE) images, with a resolution of 0.25 m/pixel, where available, to provide further morphological details on the LEHAF. However HiRISE images were not used to regionally map the mounds, because of their highly sporadic spatial distribution across the study area. THEMIS Nighttime Infrared images were consulted to gather additional information of the thermal behaviour of the target features. The image analysis was performed within ArcGIS® software using sinusoidal projections centered in the study area in order to minimize spatial distortion.

### Crater size-frequency distributions

We used the established CraterTools for ArcGIS, coupled with CraterStats to carry out standardized age estimation of the surfaces of interest^55^. Model ages were obtained by fitting the crater production function of Hartmann and Daubar^55^ to our data. By adopting this chronology system we took into account the cumulative resurfacing correction and lower diameter limits of 200 m for TPT, and of 500 m for the underlying unit, for the age estimation. Following Kneissl *et al*.^56^ we used sinusoidal map projections to avoid incorrect area size determination and considered resurfacing corrections. We delimited impact craters by identifying two opposing points on the impact-crater rim along the illumination direction where possible, because this direction provides the best contrast to correctly identify the crater rim. Due to the particular surface distribution of LEHAF within the TPT, we delimited the area representing the TPT by outlining patches of interconnected LEHAF. We did not use buffered-CSFD, because the close proximity of LEHAF patches would have led to significant buffer area overlap invalidating the method.

Absolute model ages are influenced by the size of the area considered for the CSFD analysis because of local variability and cratering patterns^57^. In accordance with the guidelines of Warner *et al*.^57^, we analyzed areas equal to and/or exceeding 1000 km^2^ to produce statistically reliable outcomes^57,58^ (~1000 km^2^ for TPT and >10000 km^2^ for the underlying unit).

### Cluster and fractal analyses

The investigation of the subsurface was performed by means of the application of cluster and fractal analyses on the entirety of the mapped LEHAF population. We mapped 2126 individual LEHAF in Arcadia Planitia, over an area of about 12000 km^2^ centered at N50° E175° fully covered by TPT. Each feature was distinguished from the neighboring one through manual identification and mapping of the summit pits. Where chains of coalescent LEHAF occurred, the summit pit identification was supported by the observation of associated moats or swellings and circumferential troughs in plan view.

We performed the spatial distribution analyses following the two-step workflow of clustering and fractal investigation of Mazzarini and Isola^29^. Clustering of features was performed to delimitate groups of LEHAF that are more likely linked to the same fracture system by applying an agglomerative hierarchical clustering method^28^ to the sinusoidal center coordinates in MINITAB® software. Each cluster subsequently underwent a self-similar clustering analysis, i.e. fractal analysis, which investigates the spatial properties of the examined objects, thus how fractures or vents fill the space^59^. This methodology, starting from the observation of point features (e.g. vents), provides insights about the possible presence of connectivity between LEHAF and a potential underlying fracture network. It allows the distinction between fracture-related and fracture-unrelated processes. Where fracture-related processes are recognized, this technique can be used to infer the extension of the connected fracture network able to drive fluids from a deep source towards the surface^27^. When the fracture network is active its spatial distribution is fractal and its self-similarity can be defined in a specific lengthscale bounded by a lower and an upper cutoff^27^. Fractal behavior is recognized when a plateau is visible in a *Δlog(C(l)/Δlog(l)) vs. log(l)* diagram where *C(l)* is the correlation sum, defined as *C(l)* = *2 N(l)/N(N − 1)* and *N(l)* are the pairs of points (i.e. the locations of the mapped central pits) whose distance is less than *l*^29,52^. The start and end of the plateau define the lower and upper cutoffs (Lco and Uco) where the upper cutoff is assumed to represent the distance between the surface and the fluid source at depth^29^. Thus, the end of the plateau (i.e.Uco) is the maximum value of the size range, which should represent the maximum extension of the fracture network connecting the reservoir(s) to the surface.

To produce the best estimate for the Uco values we adopted measures to minimize the errors. Firstly, we identified the Uco depth range corresponding to the higher values of R^2^, where R^2^ is defined as shown in equation (1)

1 $${R}^{2}=1-\,\frac{S{S}_{res}}{S{S}_{tot}}$$

where SS_res_ represents the residuals sum of squares, which is meant to be minimized in proportion to the normalization coefficient SS_tot_ (total sum of squares) in accordance to the order of magnitude involved in the calculation. The measurement error was estimated calculating the half difference between the maximum and the minimum depths where the R^2^ shows the best-fit value^27^. Secondly, in order to produce meaningful estimates, the sample size effect (i.e. the size of the population under analysis) needed to be considered. As a rule of thumb, at least 50 samples are required to extract robust parameter estimates^60^ and, we included a minimum of 309 and a maximum of 863 LEHAF per cluster. Mazzarini and Isola^27^ showed that removing a random sample of 20% of the vents from a large (i.e. >200 vents) dataset does not affect the estimation of the fractal exponent (less than 0.01% of variation) and the error introduced in the estimation of the cut-offs is less than 1–2%. In Mazzarini *et al*.^61^ the effect of uncertainties in the mapped point locations was explored by adding random errors (in the 0–100 m, 0–300 m and 0–500 m ranges) to their locations. In this test, the added errors were as high as five to twenty-five times that of the coarsest image resolution used to locate the vents. The 0–100 m errors generate fractal exponent and cut off value identical to those computed for the original dataset. In the case of 0–500 m random errors, the resulting fractal exponent is 3% higher than that computed for the original dataset, and the cut offs are very similar to those computed for the original dataset.
